# The Importance of Telerehabilitation and Future Directions for the Field

**DOI:** 10.2196/76153

**Published:** 2025-04-29

**Authors:** Sarah Munce

**Affiliations:** 1 Holland Bloorview Kids Rehabilitation Hospital Bloorview Research Institute Toronto, ON Canada; 2 Institute of Health Policy, Management and Evaluation University of Toronto Toronto, ON Canada; 3 Rehabilitation Sciences Institute University of Toronto Toronto, ON Canada

**Keywords:** JMIR Rehabilitation and Assistive Technologies, telerehabilitation, assistive technologies, access, equity, personalized care

## Abstract

During the COVID-19 pandemic, telerehabilitation was critical to providing ongoing care for people with impairments or disabilities, and it remains frequently used and popular after the pandemic. Telerehabilitation has been shown to be feasible and effective in a variety of conditions, including chronic heart failure and coronary artery disease, stroke, multiple sclerosis, and spinal cord injuries, with adverse events being rare. This editorial identifies important areas and future directions for the field, including implementation considerations in the postpandemic context, issues of access and equity, and emerging innovations and personalized care. The development and implementation of this knowledge will ensure that individuals with disabilities and impairments will continue to receive effective, safe, and person-driven care remotely.

## Importance of Telerehabilitation, Including Demonstrated Effectiveness, Feasibility, and Safety

During the COVID-19 pandemic, telerehabilitation was critical to providing ongoing care for people with impairments or disabilities [1], and it remains frequently used and popular after the pandemic. Telerehabilitation is a subset of telemedicine that uses telecommunication technologies to deliver rehabilitation services synchronously or asynchronously to patients at a distance [2]. It allows for the provision of services to patients who would otherwise be unable to access in-person services due to distance (ie, individuals living in rural and remote locations), disabilities that limit their ability to travel to in-person appointments, and the absence of caregivers who can assist with travel. Telerehabilitation comprises diagnosing, evaluating, and managing health care for individuals with physical, cognitive, or social impairment and disability [2].

Telerehabilitation has been shown to be feasible and effective in a variety of conditions, including chronic heart failure and coronary artery disease [3], stroke [4], multiple sclerosis [5], and spinal cord injuries [6], with adverse events being rare. Indeed, the convenience of telerehabilitation may lead to decreased travel expenses for participants and higher attendance rates for individuals with other life commitments [7]. According to a systematic review of randomized controlled trials on the effectiveness of telerehabilitation exercises on pain, physical function, and quality of life in individuals with physical disabilities, when compared with other interventions, there was high-quality evidence that telerehabilitation was not different to other interventions for pain (95% CI −0.4 to 0.1), physical function (95% CI −0.2 to 0.2), and quality of life (95% CI −0.1 to 0.5) in the long term [8]. Our research team recently completed a scoping review summarizing the published literature on adverse events during telerehabilitation; we determined from the 81 included studies, adverse events related to the delivery of telerehabilitation were rare, mostly characterized as mild or nonsevere [9]. This scoping review, an ongoing systematic review comparing the effect of telerehabilitation versus in-person care on adverse events, and our previous research on the experiences of clinicians using a telerehabilitation toolkit [10] have helped to inform areas of future research in telerehabilitation, which are detailed below.

## Implementation Considerations

In our study on clinician perspectives on the use of a telerehabilitation toolkit [10], our team identified a variety of implementation considerations related to telerehabilitation during the pandemic. These findings included the importance of adequate infrastructure, equipment, and space, as well as the key role of organizational and leadership support in adopting telerehabilitation and availing resources to implement telerehabilitation. A greater understanding of the safety and optimization of telerehabilitation could help influence government funding and guide policy makers, which is important given that a lack of leadership and organizational support was found to hinder the implementation of telerehabilitation. Future studies are also needed to understand the ongoing determinants of telerehabilitation implementation (ie, after the pandemic) and match these with evidence-based implementation strategies. Partnerships with patients with disabilities in co-designing telerehabilitation interventions are also needed.

## Issues of Access and Equity in Telerehabilitation

As mentioned, one of the major advantages of telerehabilitation is its ability to address geographical and logistical barriers [2]. In-person rehabilitation often requires travel to specialized centers, which can be a burden for individuals with mobility issues, in remote locations, or facing financial limitations. Telerehabilitation can mitigate these challenges as health care professionals can deliver therapy and monitor progress remotely, promoting greater inclusivity and continuity of care. Future research should focus on understanding the long-term impact of telerehabilitation on health equity, particularly among equity-seeking groups. At the same time, it is important to note that while telerehabilitation can improve access for some groups (eg, those in rural and remote locations with access to software and hardware), it can also disadvantage groups without access to these resources and limited digital literacy; these are areas of research that need exploration. Finally, in our scoping review on adverse events, we reported a lack of studies among pediatric populations. The lack of evidence could hinder the delivery of telerehabilitation to youth and is thus recommended as a future area of research.

## Emerging Innovations in Telerehabilitation and Personalized Care

In our qualitative study on clinicians’ experiences of implementing a telerehabilitation toolkit during the COVID-19 pandemic, participants described innovations that resulted from implementing telerehabilitation during the pandemic. Some of these included interprofessional assessments (ie, performed in tandem by an occupational therapist and a physical therapist), which were described as especially helpful for patients with complex needs. These interprofessional collaborations in the context of telerehabilitation should continue to be studied [10]. Relatedly, the experiences, barriers, facilitators, and preferences for the delivery of telerehabilitation should continue to be studied, as should the experiences of health care professionals delivering telerehabilitation, such as the challenges of delivering remote care safely. The use of a home pulse oximetry was also described in our study [10]. Future research should continue to explore what technologies can be integrated into telerehabilitation to improve patient and caregiver outcomes. Similarly, the use of machine learning and artificial intelligence should be leveraged in order to deliver personalized or precision care for patients undergoing rehabilitation, for example, building machine learning techniques using a variety of existing data sources to optimize goal setting based on real-time data.

## Conclusion

Telerehabilitation has been shown to be feasible and effective in a variety of conditions, with adverse events being rare [3-9]. However, it is still a burgeoning field, and this editorial has identified key areas of future research to advance its implementation and impact. Key areas of future research include understanding the long-term impact of telerehabilitation on health equity, particularly among equity-seeking groups, and the ongoing determinants of telerehabilitation implementation (ie, after the pandemic) and then matching these with evidence-based implementation strategies. Partnerships with patients with disabilities in co-designing telerehabilitation interventions are also needed, as is the need to expand research in telerehabilitation among pediatric populations. Finally, emerging innovations in telerehabilitation including interprofessional collaborations and the use of machine learning and artificial intelligence should be leveraged in order to deliver personalized or precision care for patients undergoing rehabilitation. The development and implementation of this knowledge will ensure that individuals with disabilities and impairments will continue to receive effective, safe, and person-driven care remotely. Consistent with these key areas of future research, *JMIR Rehabilitation and Assistive Technologies* has launched a call for papers [11] on “Advancing Telerehabilitation Research and Innovation.” The call for papers invites submissions in the following areas related to telerehabilitation: effectiveness and safety, implementation considerations, health equity and accessibility, innovative technologies, and pediatric rehabilitation.
